# Massive parallel RNA sequencing of highly purified mesenchymal elements in low-risk MDS reveals tissue-context-dependent activation of inflammatory programs

**DOI:** 10.1038/leu.2016.91

**Published:** 2016-06-03

**Authors:** S Chen, N A Zambetti, E M J Bindels, K Kenswill, A M Mylona, N M Adisty, R M Hoogenboezem, M A Sanders, E M P Cremers, T M Westers, J H Jansen, A A van de Loosdrecht, M H G P Raaijmakers

**Affiliations:** 1 Department of Hematology, Erasmus MC Cancer Institute, Rotterdam, The Netherlands; 2 Department of Hematology, VU University Medical Center, Amsterdam, The Netherlands; 3 Laboratory of Hematology, Department of Laboratory Medicine, Radboud University Nijmegen Medical Centre and Centre for Molecular Life Sciences, Nijmegen, The Netherlands

Myelodysplastic syndromes (MDS) have long been considered hematopoietic-cell autonomous disorders in which disease initiation and progression is exclusively driven by hematopoietic cell intrinsic genetic events. Recent experimental findings have challenged this view, implicating mesenchymal elements in the bone marrow microenvironment in disease pathogenesis. Specifically, genetic perturbation of mesenchymal cells has the ability to induce MDS and acute myeloid leukemia, establishing an experimental concept of ‘niche-induced' oncogenesis.^1, 2^ Alternatively, primary alterations in hematopoietic cells have the ability to alter mesenchymal niche components, such that niche cells facilitate disease propagation in the context of xenograft transplantation.^3^ Together, these observations challenge the view that ineffective hematopoiesis and leukemic progression is exclusively driven by hematopoietic-cell autonomous events in human MDS. Translation of experimental findings to human disease is complicated by a lack of insight in the molecular wiring of primary, non-expanded mesenchymal cells in MDS. Insights into the biology of mesenchymal elements in human MDS and other hematopoietic disorders thus far have been derived from studies investigating *ex vivo*-expanded mesenchymal cells derived from the diseased bone marrow. The hierarchic, biologic and molecular relationship between these *ex vivo*-expanded cells and their *in situ* counterparts, however, has remained largely unknown. Here we describe massive parallel transcriptome sequencing of prospectively isolated mesenchymal elements from human low-risk MDS (LR-MDS), revealing a common molecular signature, distinct from both normal and *ex vivo*-expanded cells, characterized by cellular stress and upregulation of genes encoding inflammation-associated secreted factors with established inhibitory effects on hematopoiesis.

Mesenchymal cells were prospectively FACS-sorted (fluorescence activated cell sorting) from bone marrow aspirates of LR-MDS patients (*n*=12, Supplementary Table S1) and normal controls (*n*=10) using previously established markers of primary bone marrow mesenchymal cells (Figure 1a).^4^ The frequency of CD45^−^/7AAD^−^/CD235a^−^/CD31^−^/CD271^+^/CD105^+^ mesenchymal cells in LR-MDS was not significantly different from normal bone marrow (Figure 1b) (0.019±0.0086% vs 0.022±0.0066% of mononuclear cells, *P*=0.819 by unpaired Student's *t*-test), and these cells comprised a small subset of CD45^−^/7AAD^−^/CD235a^−^ ‘niche' cells (10.41±4.086% vs 12.30±5.052%, *P*=0.771) with the major constituent being CD31^+^ endothelial cells (43.80±7.243% vs 38.28±9.424%, *P*=0.816).

RNA was extracted from highly purified mesenchymal elements and complementary DNA synthesis was performed using the SMARTer Ultra Low RNA kit for Illumina Sequencing (Clontech, Mountain view, CA, USA) (Supplementary Methods). Quality of RNA-sequencing data were shown to be similar for normal and LR-MDS-derived samples using various quality parameters including the number of aligned bases, base composition, coverage coefficient and full-length transcript coverage (from 5′ end to 3′ end) reflecting no systematic 5′-end or 3′-end bias (Supplementary Figure S1). The mesenchymal identity of CD45^−^/7AAD^−^/CD235a^−^/CD31^−^/CD271^+^/CD105^+^ cells was confirmed molecularly by whole-transcriptome analysis demonstrating significant abundance of transcripts encoding defining membrane proteins (Figure 1c), established markers of mesenchymal stem cells (Figure 1d),^4, 5^ essential ‘niche' factors governing the behavior of hematopoietic stem and progenitor cells (Figure 1e), and osteolineage markers (Figure 1f) compared with endothelial cells. Collectively, the findings demonstrate the feasibility of prospective isolation and molecular characterization of highly purified primary mesenchymal elements in LR-MDS by massive parallel transcriptome sequencing.

Principal component analysis of all transcriptomes demonstrated uniform clustering of normal mesenchymal cells, implying transcriptional homogeneity (Figure 2a). Strikingly, distinct and more heterogeneous clustering of mesenchymal transcriptomes was found in LR-MDS, revealing that these cells are transcriptionally distinct from their normal counterparts. Gene set enrichment analysis (GSEA) was subsequently performed to define the molecular networks underlying the distinct transcriptional landscape of LR-MDS. Gene sets associated with inflammatory response and cellular stress were remarkably enriched in LR-MDS (Figures 2b and c; Supplementary Table S2). Cellular stress was reflected by a reduced colony forming capacity of the CD271^+^ mesenchymal population in LR-MDS to form colonies (Figure 2d) with morphologic features reminiscent of cellular senescence (Figure 2e), as described earlier for expanded stromal cells in LR-MDS.^6, 7^ Distinct hierarchical clustering and the signatures of cellular stress were not age-dependent, as these signatures remained statistically significant when examined in an age-matched sub-cohort of patients and controls (Supplementary Figure S2). Together, the data indicate that mesenchymal cells in LR-MDS are molecularly and functionally distinct from their normal counterparts, characterized by cellular stress, reflected by a reduced *ex vivo* capacity to form fibroblast colonies.

Thus far, molecular and biologic insights into the role of mesenchymal cells in the pathogenesis of human MDS have been derived from studies using *ex vivo*-expanded and plastic adherent stromal cells. The molecular relationship between these expanded cells and their *in situ* mesenchymal counterparts has remained largely unknown. Elucidation of the transcriptome of mesenchymal elements in the MDS marrow allows us to compare our transcriptional data with sequencing data obtained from expanded cells in an age-matched cohort of LR-MDS published earlier (Supplementary Figure S3).^3^

Comparison of FDR-significant differentially expressed transcripts between the two data sets demonstrated limited overlap (Figure 2f), suggesting distinct molecular wiring between the two mesenchymal cell sources. To obtain insight into the biologic processes underlying differential gene expression, GO (gene ontologies) term analysis was performed focusing on cellular biologic processes. To correct for potential experimental differences affecting fragments per kilobase of exon per million fragments mapped (FPKM) values, we normalized expression of all genes in LR-MDS to the expression of the controls in the respective data sets as detailed in the Supplementary Methods section. Normalized expression was subsequently used to perform GO term analysis and GSEA, comparing sorted with expanded cells.

25 GO terms were significantly (FDR<0.25) enriched in primary CD271^+^ mesenchymal cells (whereas no signatures were enriched in the *ex vivo*-expanded mesenchymal cells), many of which (8/25) reflected response to external stimuli, chemokine activity and immune regulation (Figure 2g). Transcript abundance analysis in CD271^+^ cells in comparison with their normal counterparts indeed revealed a significant upregulation of numerous cytokines (Supplementary Table S3), including a large number of inflammatory factors such as interleukin(IL)-6 and IL-8, and a wide variety of factors previously demonstrated to be negative regulators of hematopoiesis, in particular, erythropoiesis and B-lymphopoiesis—cell lineages that are typically affected in LR-MDS (Supplementary Table S3).

To obtain insight into the molecular pathways underlying the biologic processes identified, transcriptional network analysis (GSEA) was performed. This identified 504 gene sets that were significantly (FDR<0.25) enriched in primary-sorted LR-MDS stromal cells, whereas 16 signatures were enriched in expanded LR-MDS stromal cells. Again, gene signatures related to inflammation and cellular stress were enriched in CD271^+^ cells with a remarkable abundance of signatures related to epidermal growth factor (EGF), transforming growth factor beta (TGFβ) and tumor necrosis factor (TNF) signaling (Supplementary Figure S4, Supplementary Table S4).

Collectively, the data comprise, to our knowledge, the first comprehensive transcriptional network analysis of highly purified mesenchymal elements directly isolated from the marrow in human hematopoietic disease. They support the view that these cells are intricately implicated in MDS disease pathogenesis, stressing the relevance of considering the tissue context in generating a comprehensive understanding of the disease. The data further support the notion that inflammatory signaling is an important pathophysiologic factor in LR-MDS and implicate the mesenchyme in this process. Finally, the data complement findings derived from *ex vivo* stromal cells in this disease revealing preferential overexpression of inflammatory pathways and secreted factors in FACS-purified CD271^+^ cells. This likely reflects active cross talk with other cellular elements within the inflammatory bone marrow environment in LR-MDS,^8^ eliciting or maintaining these transcriptional programs, which may not be fully appreciated in *ex vivo* cultures.

The finding that secretory programs implicated in negative regulation of hematopoiesis are activated in CD271^+^ cells, may be of particular relevance, given their close anatomical proximity with CD34^+^ cells,^9^ potentially harboring the MDS initiating population.^10^ The data warrant future investigations unraveling the signaling between cellular elements in the MDS marrow driving these secretory programs. We expect that elucidation of the transcriptome of highly purified mesenchymal elements in MDS will thus be a valuable resource to the community, instructing the validation and discovery of novel pathophysiologic factors and putative therapeutic targets.^11^

## Figures and Tables

**Figure 1:**
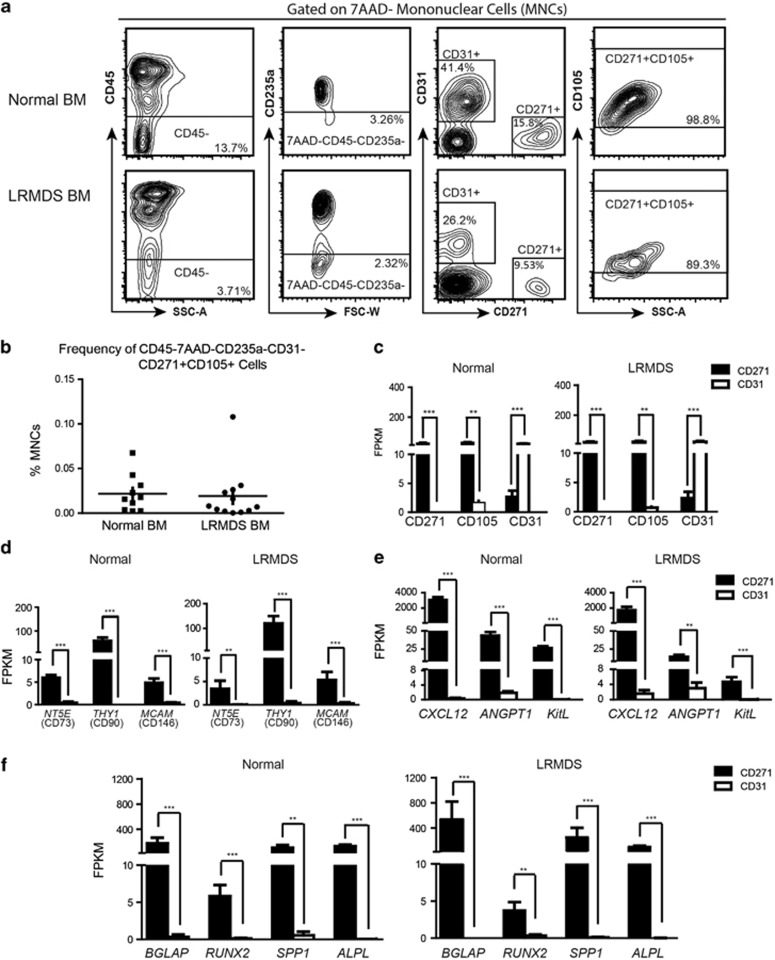
Prospective isolation and molecular characterization of mesenchymal cells in LR-MDS. (**a**) Gating strategy to identify and isolate 7AAD^−^/CD45^−^/CD235a^−^/CD271^+^/CD105^+^ mesenchymal cells. (**b**) Frequency of mesenchymal cells in normal and MDS samples. (**c**–**f**) Transcriptional validation of the mesenchymal identity of 7AAD^−^/CD45^−^/CD235a^−^/CD271^+^/CD105^+^ cells, revealing differential expression in comparison with endothelial subsets of (**c**) defining cell surface markers (CD271, CD105 and CD31), (**d**) known mesenchymal markers (CD73, CD90 and CD146), (**e**) established hematopoiesis-supporting cytokines (*CXCL12, ANGPT1 and KITL*) and (**f**) bone lineage markers (*BGLAP, RUNX2, SPP1* and *ALPL*). BM, bone marrow; FPKM, fragments per kilobase of exon per million fragments mapped; FSC-W, forward scattered light-width; SSC-A, side scattered light-area; 7AAD (7-aminoactinomycin D); CD45 (*PTPRC*: protein tyrosine phosphatase, receptor type, C); CD235a (Glycophorin A); CD271 (*NGFR*: nerve growth factor receptor); CD105 (*ENG*: endoglin); CD31 (*PECAM-1*: platelet/endothelial cell adhesion molecule-1). CD73 (*NT5E*: ecto-5′-nucleotidase); CD90 (*THY1*: Thy-1T-cell antigen); CD146 (*MCAM*: melanoma cell adhesion molecule); *CXCL12* (stromal cell-derived factor 1); *ANGPT1* (angiopoietin 1); *KITL* (KIT ligand); *BGLAP* (osteocalcin); *RUNX2* (runt-related transcription factor 2); *SPP1* (osteopontin); *ALPL* (alkaline phosphatase, liver/bone/kidney). (**c**–**f**) Normal samples (*n*=10); MDS samples (*n*=12). Black bar: CD271^+^ mesenchymal cells; white bar: CD31^+^ endothelial cells. FDR, false discovery rate. **FDR<0.01; ***FDR<0.001.

**Figure 2:**
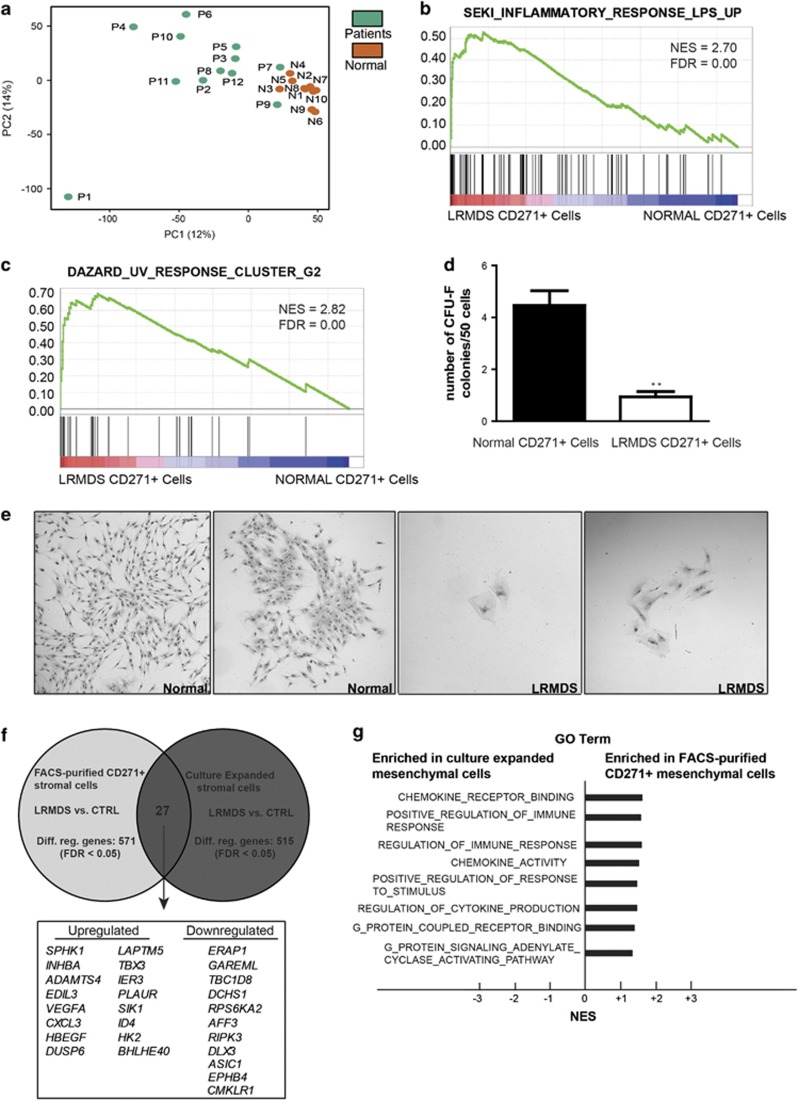
Mesenchymal cells in LR-MDS display a distinct molecular signature characterized by cellular stress and inflammation. (**a**) Principal component analysis (PCA) on the transcriptomes of normal and LR-MDS mesenchymal cells. Patient numbers in **a** refer to LR-MDS patient IDs (Supplementary Table S1). (**b**) Example of GSEA plot revealing inflammatory response in the mesenchymal cells from LR-MDS. (**c**) Representative GSEA plot demonstrating deregulation of the gene set associated with cellular stress in response to UV in LR-MDS mesenchymal cells. Gene set size, NES and FDR values of each gene set is listed. (**d**) Number of CFU-F colonies formed by normal (*n*=3) or LR-MDS (*n*=3) CD271^+^ mesenchymal cells. (**e**) Representative images of cell clusters and colonies formed by mesenchymal cells from healthy control (left panel) and LR-MDS patients (right panel). (**f**) Comparison of significantly differentially expressed genes in FACS-purified CD271^+^ versus culture-expanded mesenchymal cells in LR-MDS. The total number of differentially regulated transcripts in each data set is indicated and the overlapping differentially regulated genes in the two data sets are listed. (**g**) Biologic processes significantly enriched (FDR<0.25) in FACS-purified CD271^+^ LR-MDS mesenchymal cells in comparison with expanded stromal cells defined by GO term analysis.**P<0.01. CFU-F, colony-forming unit - fibroblast; FDR, false discovery rate; NES, normalized enrichment score; UV, Ultraviolet.
