# Supplementary material for: Massive parallel RNA sequencing of highly purified mesenchymal elements in low-risk MDS reveals tissue-context-dependent activation of inflammatory programs
Source: Leukemia. 2016 Jun 3;30(9):1938–42. doi: 10.1038/leu.2016.91 (PMC5240018; doi:10.1038/leu.2016.91)
Supplement: Supplementary Information [file leu201691x1.docx]

**Supplemental data ­**

**Supplementary material and methods**

**Patient and healthy donor bone marrow samples**

Patient characteristics (median age: 65, range 38-80) are shown in Table S1. Control marrow was obtained from donors for allogeneic transplantation (median age: 45, range 35-61), after written informed consent. The use of human samples was approved by the Institutional Review Board of the Erasmus Medical Center, the Netherlands, in accordance with the declaration of Helsinki.

**Flow cytometry analysis**

For cell sorting, bone marrow from patients and normal donors were stained on ice in the dark with the following antibodies using optimized dilutions: CD45-PE-Cy7 (1:200), CD235a-BV421-A (1:100), CD271-PE (1:100), CD105-APC (1:50), CD31-APC-CY7 (1:50). The indicated populations of interest were sorted using a FACS ARIAIII Cell Sorter (BD Biosciences). Dead cells were gated out using 7AAD (Stem-Kit Reagents) after mononuclear cell selection and doublets exclusion.

The cells were directly sorted in 800μl Trizol (Ambion) for RNA isolation. RNAse free non-stick micro-tubes (Ambion) were used to prevent pre-digestion of RNA.

**RNA extraction and RNA quality control**

Total sample RNA isolation was performed according to the standard protocol of RNA isolation with Trizol and GenElute LPA (Sigma). The RNA pellet was resuspended in 7.5μl of RNAse free water (Qiagen) and quality and quantity of the total RNA was checked on a 2100 Bio-analyzer (Agilent) using the Agilent RNA 6000 Pico Kit.

**RNA sequencing and gene expression profiling**

SMARTer Ultra Low RNA Kit (Clonetech) for Illumina Sequencing was used to prepare the cDNA based on the manufacture’s recommendation. Similar quantities of total RNA from each sample were used as starting material for the SMARTer procedure. cDNA preparation steps were performed according to the user manual in a PCR-clean room to avoid contamination. The Agilent 2100 Bio-analyzer and the High Sensitivity DNA kit were applied to determine the quantity and quality of the cDNA production. Once the cDNAs were obtained, the subsequent library preparation steps, sequencing and alignments were performed as previously described.^(^[^1^](#_ENREF_1)^,^ [^2^](#_ENREF_2)^)^ In brief, prior to sequence alignment, the SMARTer adapters were trimmed using the cutadapt program.^(^[^3^](#_ENREF_3)^)^ The resulting sequences were aligned to the human RefSeq transcriptome using TopHat2.^(^[^4^](#_ENREF_4)^)^ Sequences that could not be aligned to the RefSeq transcriptome were aligned to the reference genome (build hg19). Normalization and quantification was performed using Cufflinks.^(^[^5^](#_ENREF_5)^)^ The resulting gene expression values are measured as FPKM (Fragments per kilobase of exon per million fragments mapped). Fragment counts were determined per gene with HTSeq-count, utilizing the strict intersection option, and subsequently used for differential expression analysis using the DESeq2^(^[^6^](#_ENREF_6)^)^ package, with standard parameters, in the R environment. Multiple testing correction was performed with the Benjamini-Hochberg procedure to control the False Discovery Rate (FDR). Principle component analysis was performed on the fragment counts using the R environment. Finally gene set enrichment analysis (GSEA) was performed on the FPKM values using the curated C2 collection of gene sets within MSigDB.^(^[^7^](#_ENREF_7)^)^

**Comparison between molecular data from primary and *ex vivo* expanded mesenchymal cells**

We performed a direct comparison of transcriptional data obtained from FACS-isolated CD271^+^ mesenchymal cells to transcriptional data obtained by RNAseq from culture-expanded stromal cells from an age-matched cohort of LR-MDS patients reported earlier.^(^[^8^](#_ENREF_8)^)^ BAM files containing the molecular data of *ex vivo* expanded stromal cells derived from LR-MDS patients (n=5) and normal controls (n=3)^(^[^8^](#_ENREF_8)^)^ were obtained from the European Genome-Phenome Archive data base (EGAS00001000716). Gene expression values quantified by the FPKM statistic were determined by Cufflinks as previously described (*vide supra*). Differential expression analysis was performed using the DESeq2 package.

To allow direct comparison of datasets and correct for potential technical/experimental variation affecting FPKM values, for both datasets, average gene FPKM values of MDS samples were normalized to average FPKM values observed in the normal control samples (FPKM MDS/FPKM control)and the normalized (fold-change) value subjected to GO term and GSEA.^(^[^7^](#_ENREF_7)^)^

**CFU-F assay**

Live CD45^-^/CD235a^-^/CD271^+^ mesenchymal cells from bone marrow samples of LR-MDS patients (n=3) and normal controls (n=3) were sorted using a FACSARIA III cell sorter. On average, 20 cells per 0.32 cm^2^ were plated in ɑMEM supplemented with 20% fetal bovine serum and 1% penicillin, streptomycin. On day 14, dishes were fixed and stained with Giemsa, and CFU-F colonies were counted as previously described.^(^[^9^](#_ENREF_9)^)^ Images of colonies and individual cell clusters were acquired on a Leica SP5 confocal laser scan microscope using a 40x objective. Images were analyzed using ImageJ software.

**Supplementary reference**

1. Groschel S, Sanders MA, Hoogenboezem R, de Wit E, Bouwman BA, Erpelinck C, et al. A single oncogenic enhancer rearrangement causes concomitant EVI1 and GATA2 deregulation in leukemia. Cell. 2014;157(2):369-81.

2. Zambetti NA, Bindels EM, Van Strien PM, Valkhof MG, Adisty MN, Hoogenboezem RM, et al. Deficiency of the ribosome biogenesis gene Sbds in hematopoietic stem and progenitor cells causes neutropenia in mice by attenuating lineage progression in myelocytes. Haematologica. 2015;100(10):1285-93.

3. Marcel M. Cutadapt removes adapter sequences from high-throughput sequencing reads. EMBnet journal. 2011;17:10-2.

4. Kim D, Pertea G, Trapnell C, Pimentel H, Kelley R, Salzberg SL. TopHat2: accurate alignment of transcriptomes in the presence of insertions, deletions and gene fusions. Genome Biol. 2013;14(4):R36.

5. Trapnell C, Roberts A, Goff L, Pertea G, Kim D, Kelley DR, et al. Differential gene and transcript expression analysis of RNA-seq experiments with TopHat and Cufflinks. Nat Protoc. 2012;7(3):562-78.

6. Anders S, Huber W. Differential expression analysis for sequence count data. Genome Biol. 2010;11(10):R106.

7. Subramanian A, Tamayo P, Mootha VK, Mukherjee S, Ebert BL, Gillette MA, et al. Gene set enrichment analysis: A knowledge-based approach for interpreting genome-wide expression profiles. P Natl Acad Sci USA. 2005;102(43):15545-50.

8. Medyouf H, Mossner M, Jann JC, Nolte F, Raffel S, Herrmann C, et al. Myelodysplastic cells in patients reprogram mesenchymal stromal cells to establish a transplantable stem cell niche disease unit. Cell Stem Cell. 2014;14(6):824-37.

9. Tormin A, Li O, Brune JC, Walsh S, Schutz B, Ehinger M, et al. CD146 expression on primary nonhematopoietic bone marrow stem cells is correlated with in situ localization. Blood. 2011;117(19):5067-77.

10. Kim SJ, Letterio J. Transforming growth factor-beta signaling in normal and malignant hematopoiesis. Leukemia. 2003;17(9):1731-7.

11. Broxmeyer HE, Cooper S, Hangoc G, Kim CH. Stromal cell-derived factor-1/CXCL12 selectively counteracts inhibitory effects of myelosuppressive chemokines on hematopoietic progenitor cell proliferation in vitro. Stem Cells Dev. 2005;14(2):199-203.

12. Dimicoli S, Wei Y, Bueso-Ramos C, Yang H, Dinardo C, Jia Y, et al. Overexpression of the toll-like receptor (TLR) signaling adaptor MYD88, but lack of genetic mutation, in myelodysplastic syndromes. PLoS One. 2013;8(8):e71120.

13. Nishihara T, Ohsaki Y, Ueda N, Koseki T, Eto Y. Induction of apoptosis in B lineage cells by activin A derived from macrophages. J Interferon Cytokine Res. 1995;15(6):509-16.

14. Lambert MP, Rauova L, Bailey M, Sola-Visner MC, Kowalska MA, Poncz M. Platelet factor 4 is a negative autocrine in vivo regulator of megakaryopoiesis: clinical and therapeutic implications. Blood. 2007;110(4):1153-60.

15. Bruns I, Lucas D, Pinho S, Ahmed J, Lambert MP, Kunisaki Y, et al. Megakaryocytes regulate hematopoietic stem cell quiescence through CXCL4 secretion. Nat Med. 2014;20(11):1315-20.

**Supplemental tables and figures**

**Table S1. Patient characteristics**

**Supplemental Table 1**. **Patient characteristics.** (A) Bone marrow was obtained at entry of a prospective clinical trial (HOVON89, Eudract nr. 2008-002195-10). The patient ID, age, WHO category (MDS WHO classification 2008), cytogenetic abnormalities, percentage of blasts in the bone marrow, the International Prognostic Scoring System for MDS (IPSS) and genetic aberrations of each patient are listed above (n=12). (B) Age and gender of each normal control sample is listed.

**Supplemental Table 2. Enrichment of transcriptional signatures reflecting inflammatory response and cellular stress in mesenchymal cells from LR-MDS patients.**


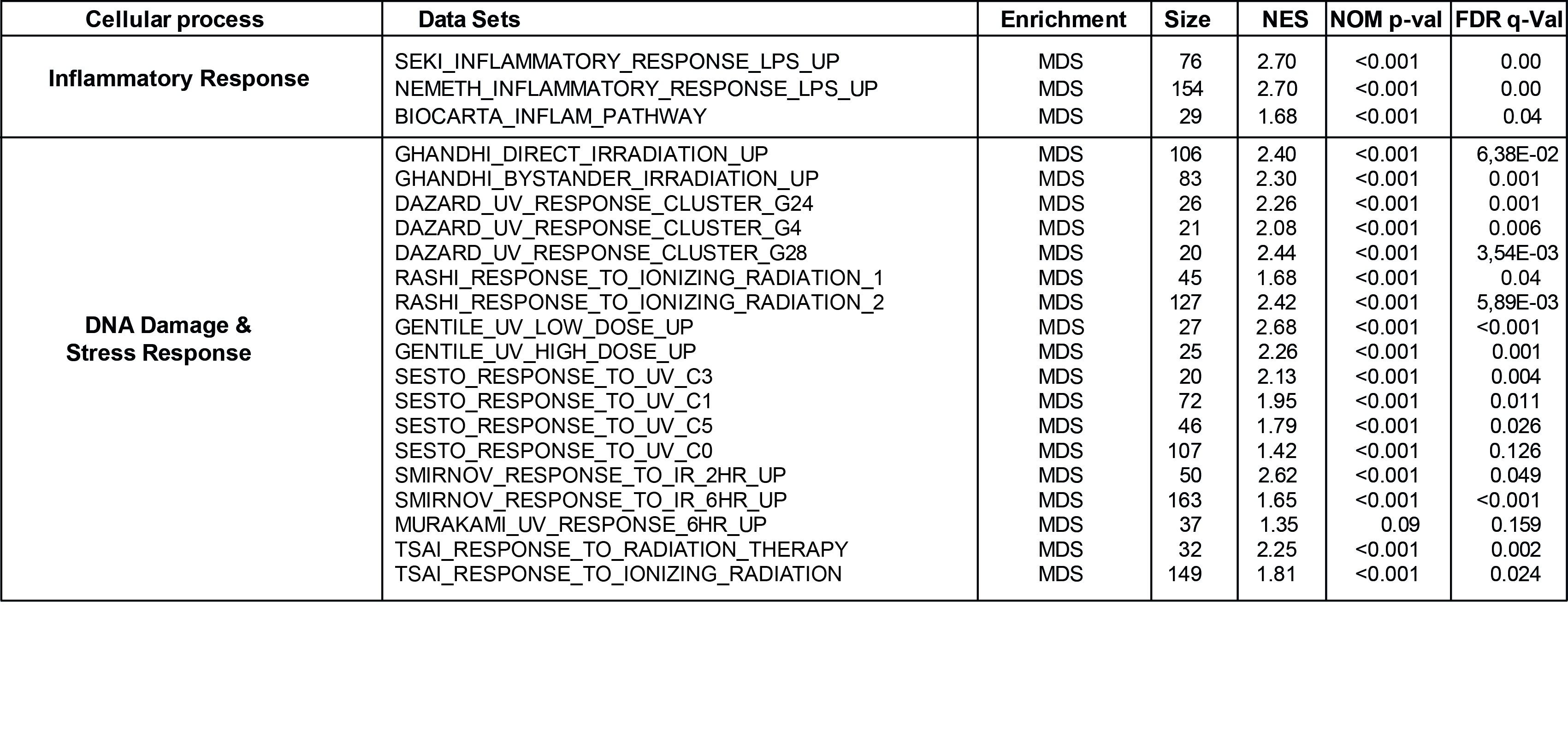
The size of each individual gene set, normalized enrichment score (NES), nominal p-value and false discovery rate (FDR) values are as listed.

**Supplemental Table 3. Differential expression of secreted factors and cytokines in primary LR-MDS mesenchymal cells**


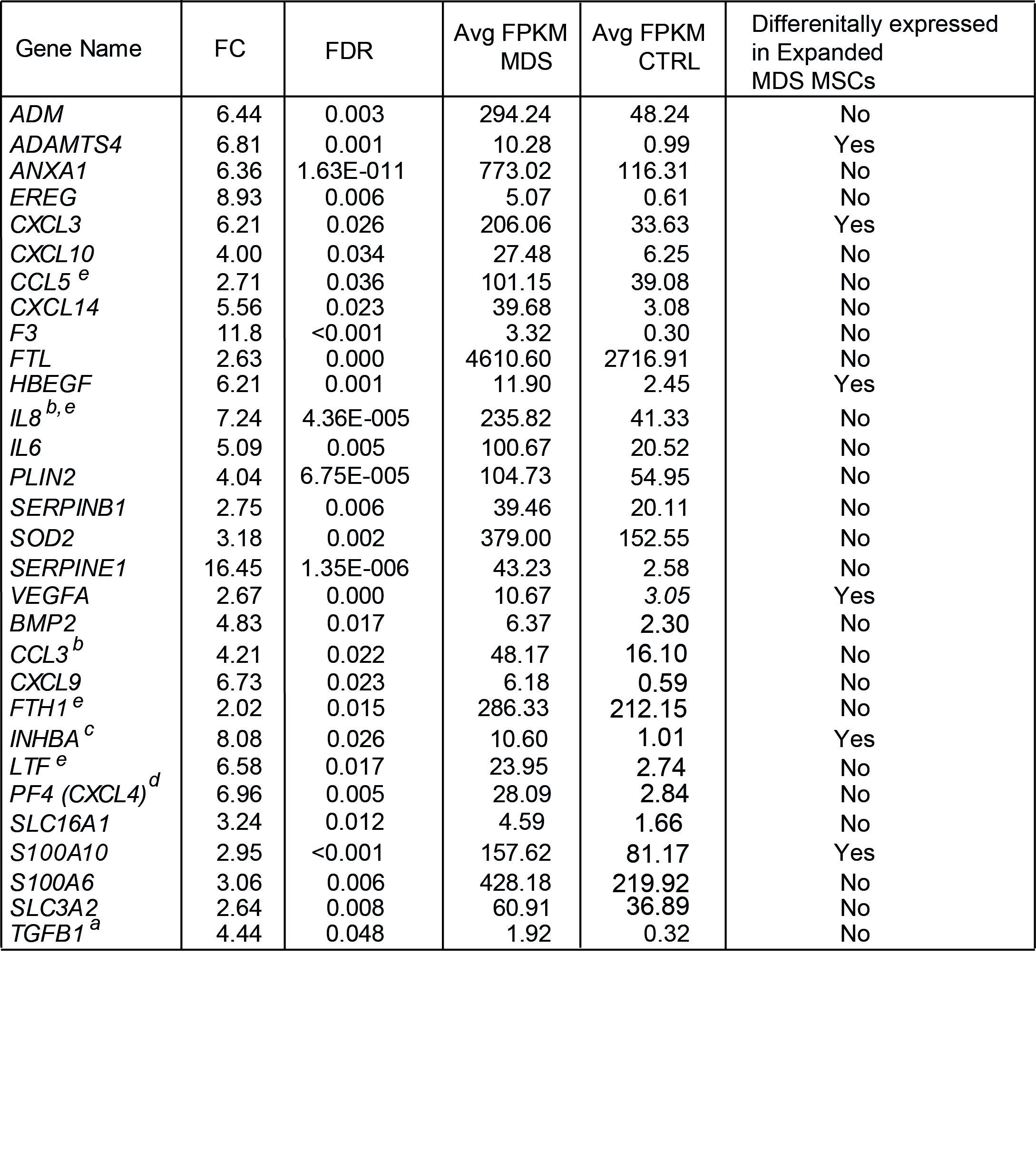


Differential expression of different cytokines in primary LR-MDS mesenchymal cells comparing to the normal counterparts are listed. This list includes bona fide inflammatory cytokines known to be negative regulators of hematopoiesis (a), erythropoiesis (b), B-lymphopoiesis (c), megakaryopoiesis (d) and with myelosuppressive effects (e).^(^[^10-15^](#_ENREF_10)^)^ FC: Fold change; FDR: false discovery rate. Fold change, FDR significance and average FPKM value of each gene are as listed. FC: Fold change; FDR: false discovery rate.

**Supplemental Table 4. Signatures that are associated with stress, inflammation, TNF and EGF signaling within the top 100 signatures enriched in primary LR-MDS mesenchymal cells in comparison to culture expanded stromal cells**

**
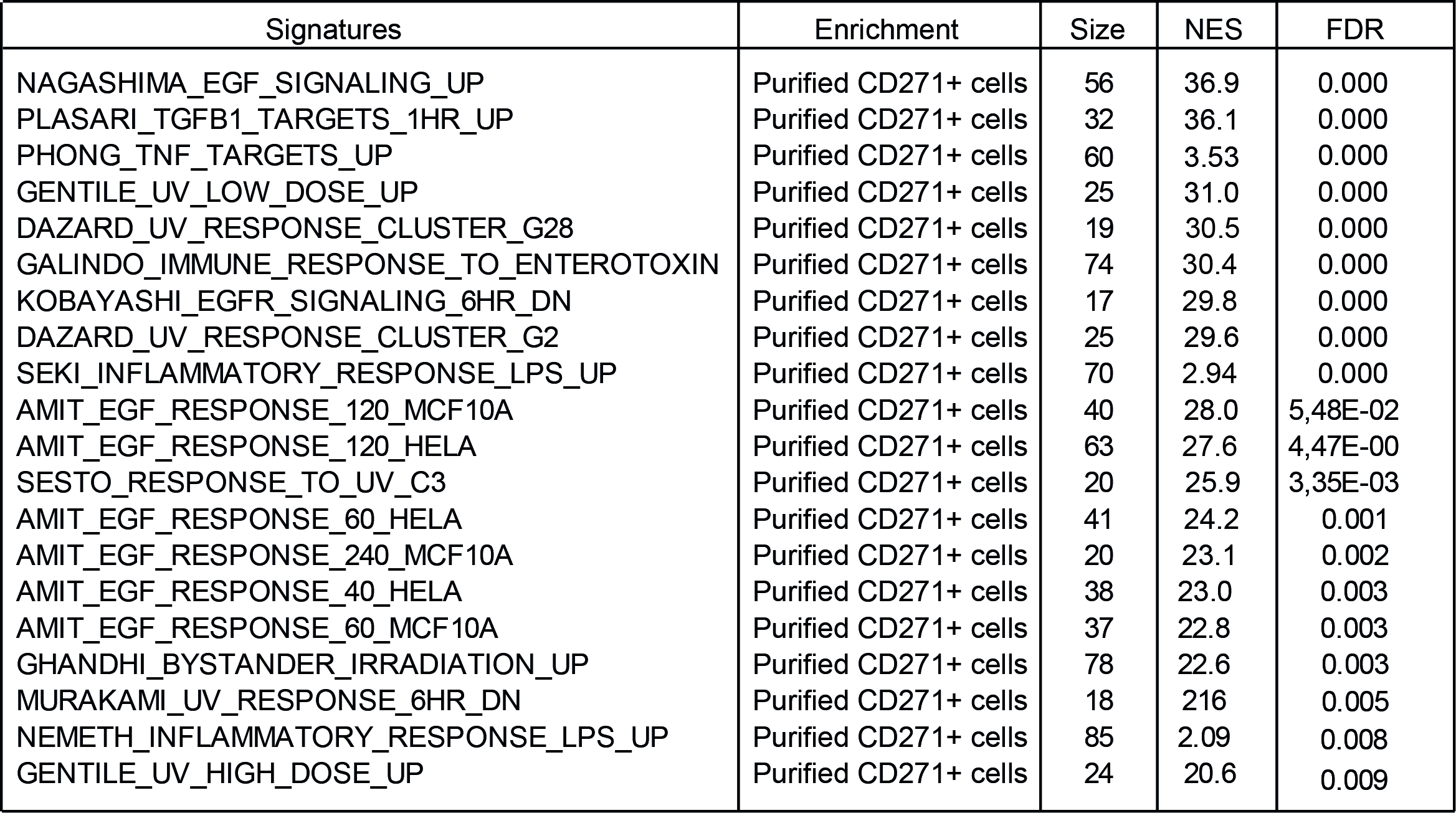
**

**Figure S1**

**
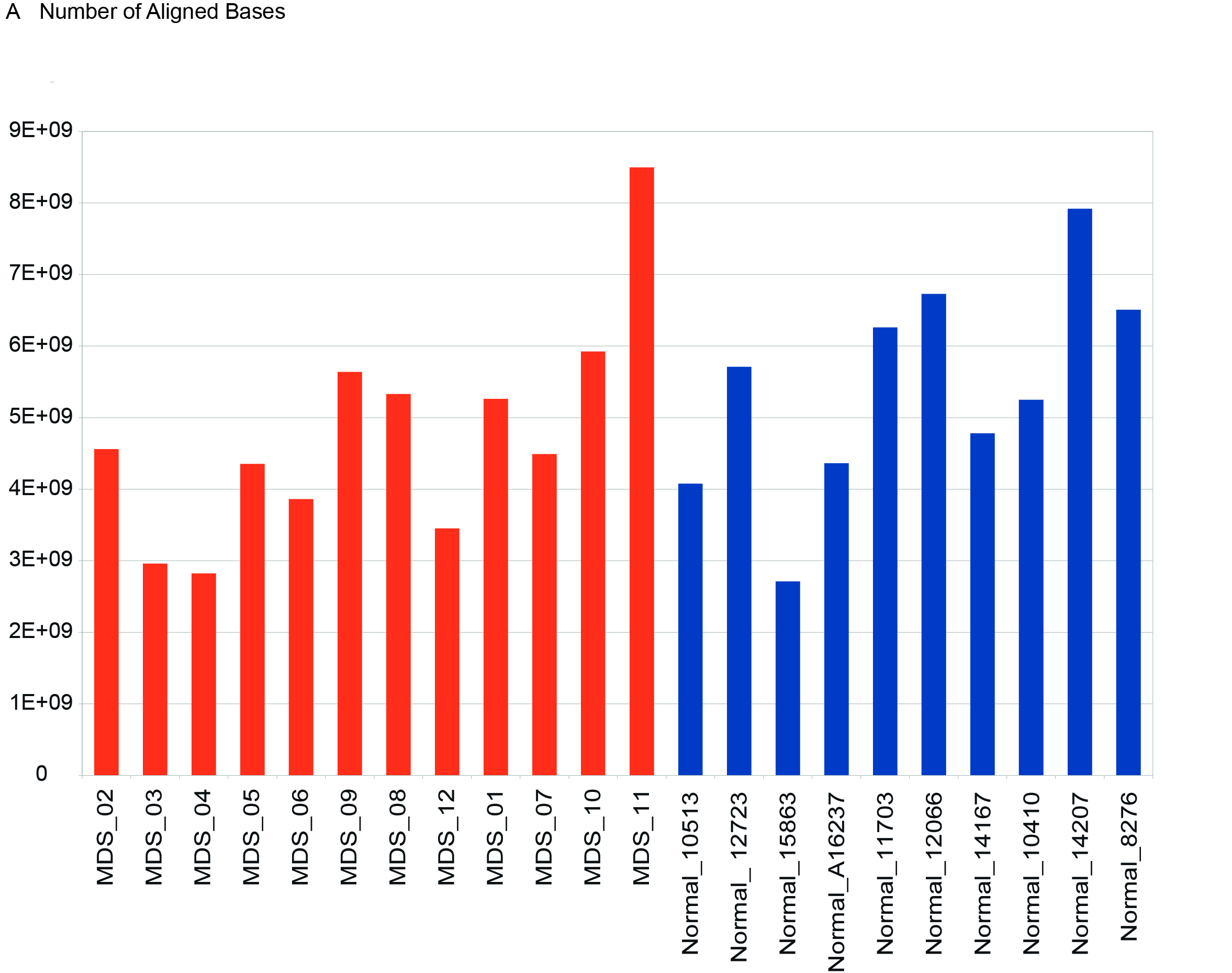
**

**
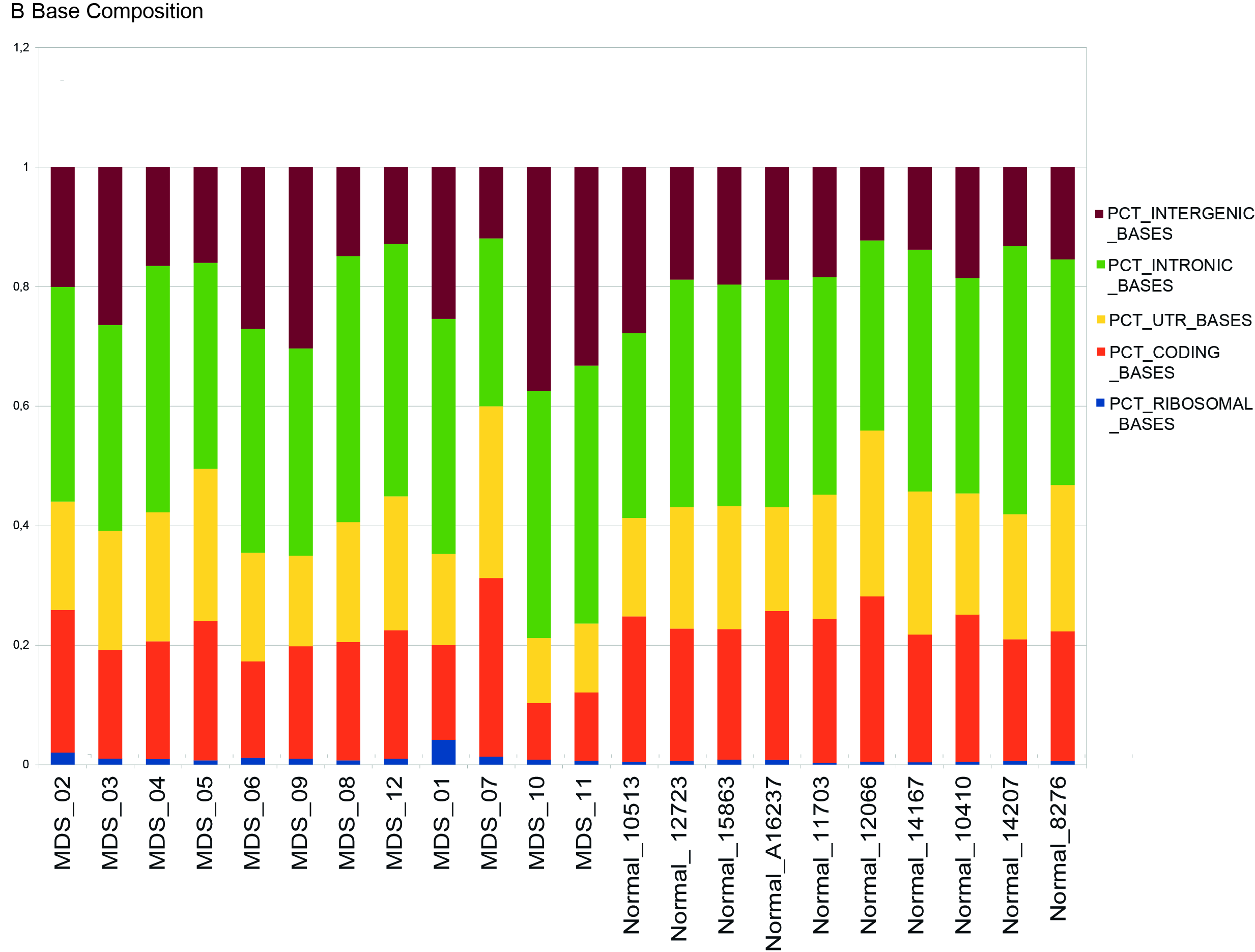
**

**
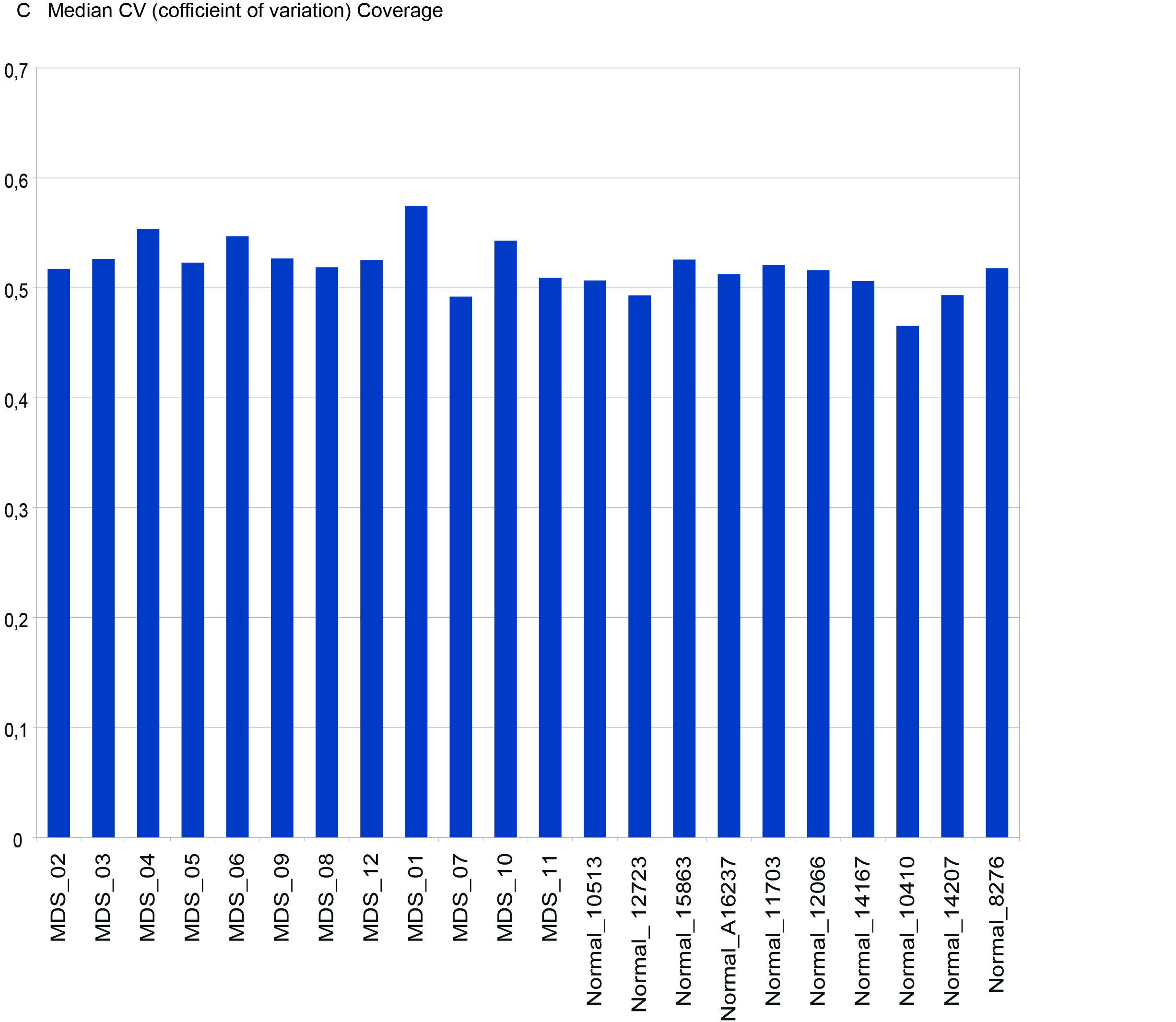
**

**
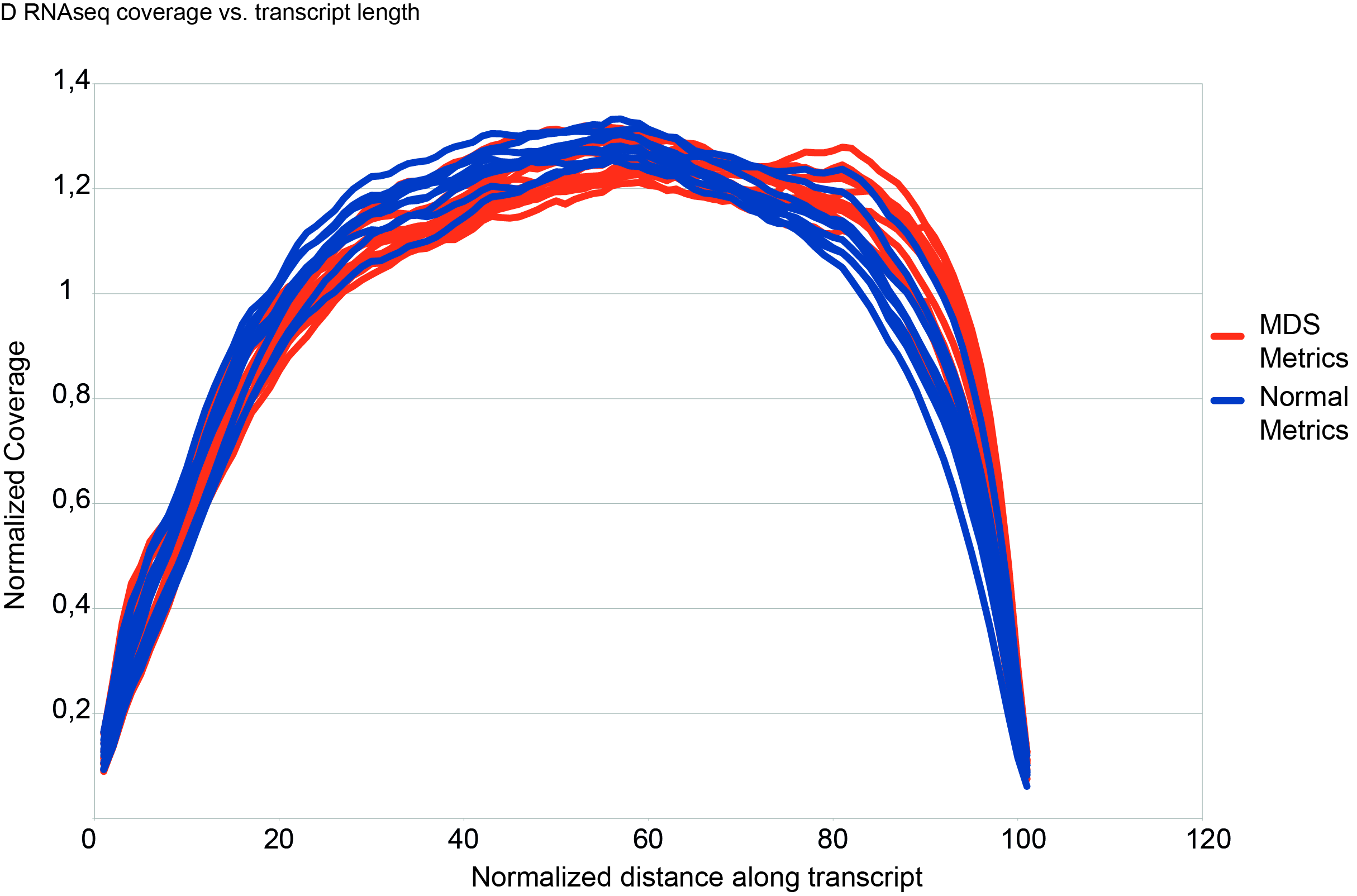
**

**Supplemental Figure 1. Comparable quality of RNA sequencing data obtained from MDS and normal mesenchymal cells.** (A) Number of aligned bases were calculated using aligned data of MDS (orange bars) *vs.* normal (blue bars) stromal samples. (B) Base composition including ribosomal bases (blue), coding bases (orange), UTR bases (yellow), intronic bases (green) and intergenic bases (red) is constructed for both MDS and normal stromal cell aligned sequencing data. (C) Median coefficient of variation (CV) value was computed indicating the level of coverage for LR-MDS and normal stromal cell aligned sequencing data. (D) Comparison of the transcript coverage from LR-MDS CD271^+^ cells and control CD271^+^ cells. Presented here are superimposed plots displaying the average read coverage from the total RNA of each sample library. The x-axis represents gene length normalized to 100%, where 0 is the 5’-end and 100 is the 3’end of each transcript.

**Figure S2**

**
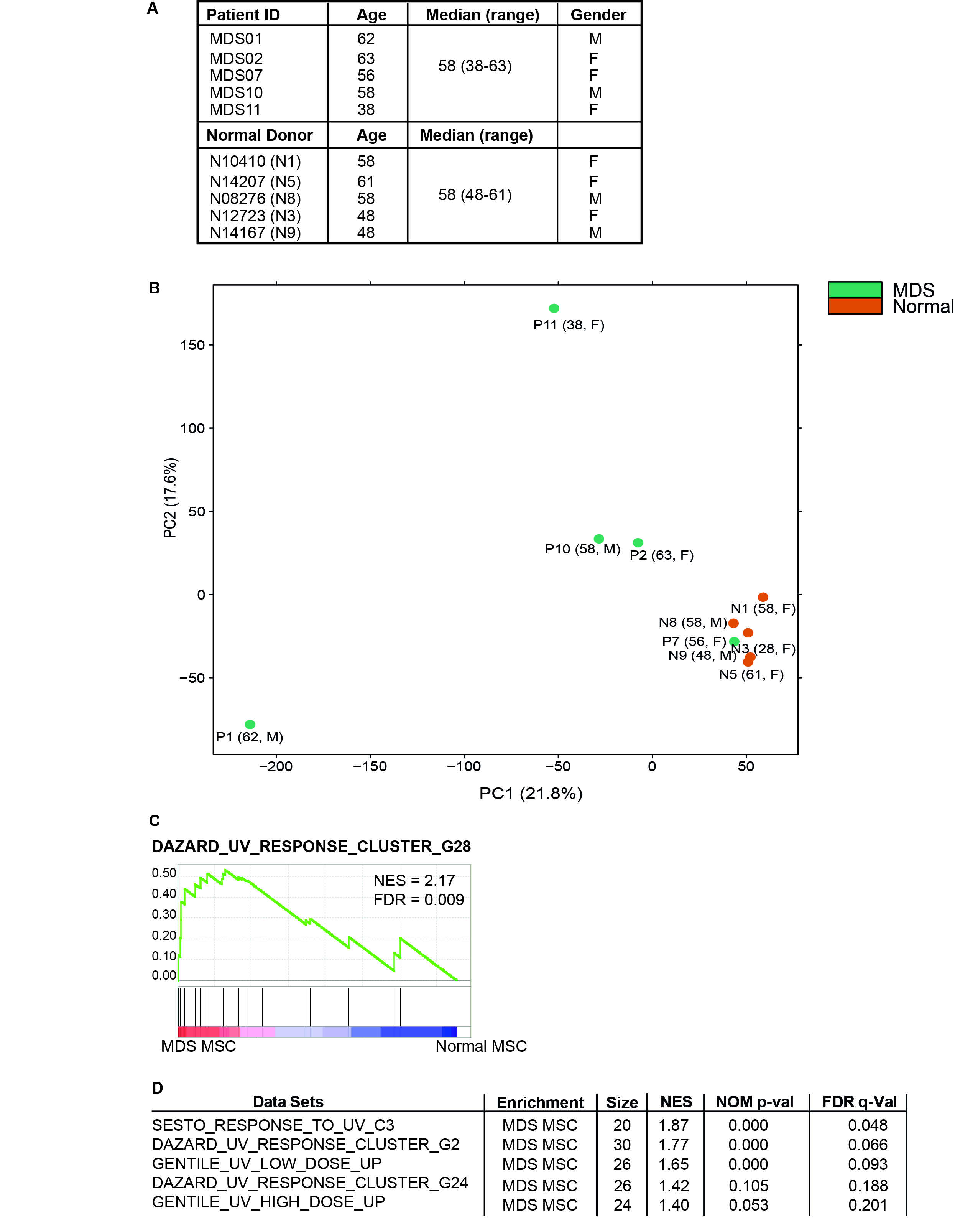
**

**Supplemental Figure 2. Molecular signature of mesenchymal cells from LR-MDS patients compared to age-matched controls.** (A) Patient and control characteristics including median age and gender. (B) PCA. (Normal: orange; patient: green). The age and gender of each sample is indicated in the plot. (C) Representative GSEA plot indicating enriched stress response in LR-MDS mesenchymal cells compared to age-matched controls. NES and FDR value of each signature are listed. (D) List of GSEA signatures reflecting cellular stress in LR-MDS stromal cells in the age-matched cohort. GSEA: gene sets enrichment analysis. NES: normalized enrichment score. FDR: false discovery rate.

**Figure S3**

**
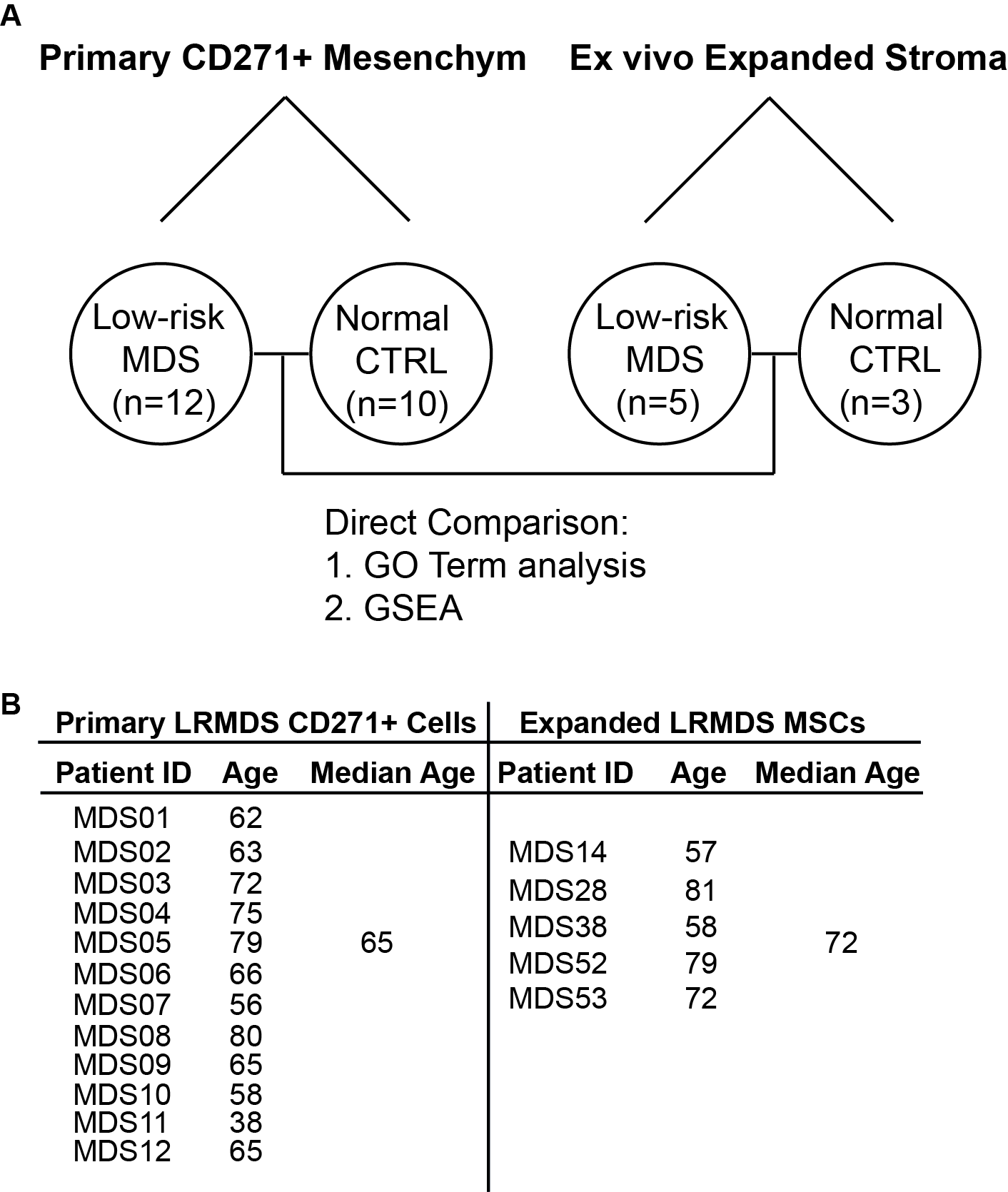
**

**Supplemental Figure 3. Comparison of molecular profile between primary and expanded LR-MDS stromal cells^(^**[**^8^**](#_ENREF_8)**^)^** (A) Scheme illustrating the strategy of directly comparing RNA-seq data from primary mesenchymal cells to RNA-seq data obtained from culture expanded stromal cells in LR-MDS. (B) Age distribution of LR-MDS patients for the primary and expanded stromal cell data sets. (C) Gene sets differentially expressed (FDR< 0.25) in primary CD271^+^ cells or *ex vivo* expanded MSCs from patients with LR-MDS.

**Figure S4**


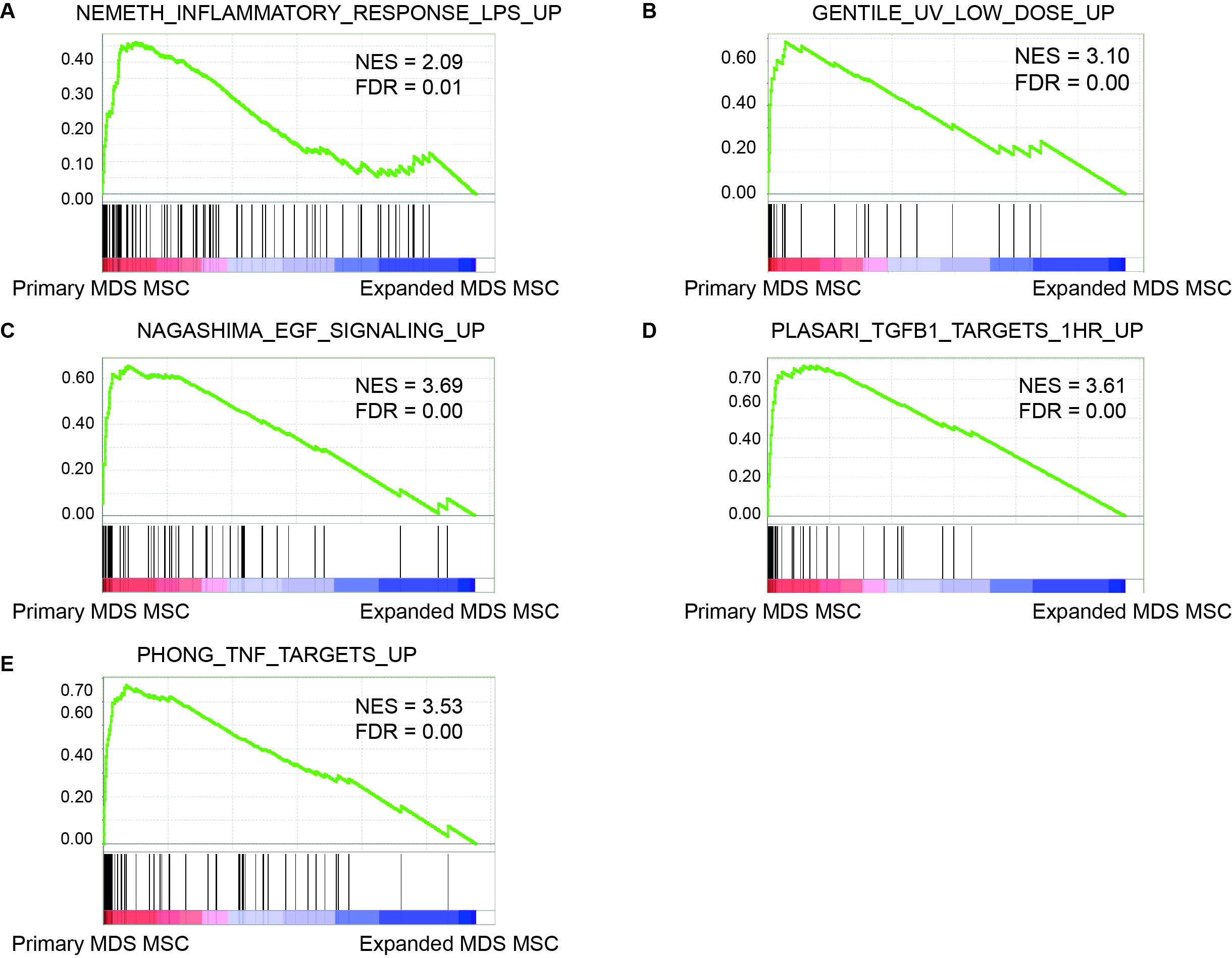


**Supplemental Figure 4. Gene signatures of inflammation and stress enriched in primary LR-MDS mesenchymal cells in comparison to expanded stromal cells.** Representative GSEA plot demonstrating enrichment of gene sets associated with inflammation (A), stress (B) and EGF, TGFß, TNF signaling (C-E) in primary LR-MDS mesenchymal cell comparing to expanded stromal cells. These plots are associated with Supplemental Table S4.
